# Glutamate at the Vertebrate Neuromuscular Junction: From Modulation to Neurotransmission

**DOI:** 10.3390/cells8090996

**Published:** 2019-08-28

**Authors:** Maria Nicol Colombo, Maura Francolini

**Affiliations:** Department of Medical Biotechnology and Translational Medicine–Via Vanvitelli, Università degli Studi di Milano, 32-20129 Milan, Italy

**Keywords:** neuromuscular junction, glutamate, acetylcholine, neurotransmitter, receptor, transporter

## Abstract

Although acetylcholine is the major neurotransmitter operating at the skeletal neuromuscular junction of many invertebrates and of vertebrates, glutamate participates in modulating cholinergic transmission and plastic changes in the last. Presynaptic terminals of neuromuscular junctions contain and release glutamate that contribute to the regulation of synaptic neurotransmission through its interaction with pre- and post-synaptic receptors activating downstream signaling pathways that tune synaptic efficacy and plasticity. During vertebrate development, the chemical nature of the neurotransmitter at the vertebrate neuromuscular junction can be experimentally shifted from acetylcholine to other mediators (including glutamate) through the modulation of calcium dynamics in motoneurons and, when the neurotransmitter changes, the muscle fiber expresses and assembles new receptors to match the nature of the new mediator. Finally, in adult rodents, by diverting descending spinal glutamatergic axons to a denervated muscle, a functional reinnervation can be achieved with the formation of new neuromuscular junctions that use glutamate as neurotransmitter and express ionotropic glutamate receptors and other markers of central glutamatergic synapses. Here, we summarize the past and recent experimental evidences in support of a role of glutamate as a mediator at the synapse between the motor nerve ending and the skeletal muscle fiber, focusing on the molecules and signaling pathways that are present and activated by glutamate at the vertebrate neuromuscular junction.

## 1. Role of Glutamate as Modulator of Cholinergic Transmission and Plasticity at the Skeletal Neuromuscular Junction of Vertebrates

Acetylcholine (ACh) is the principal neurotransmitter at the vertebrate neuromuscular junction (NMJ), however since the discovery that motoneurons and presynaptic terminals of rodent endplates from the hindlimb muscles extensor digitorum longus (EDL) and soleus are positive for glutamate labelling [1,2], it has been proposed that glutamate may participate in modulating cholinergic transmission at the NMJ of vertebrates.

A role of glutamate as modulator of cholinergic transmission and plasticity was initially suggested by the observation that its concentration was higher in the fast twitch EDL than in the slow twitch soleus muscle NMJ [2], and by more recent observations showing that glutamate immunoreactivity was higher among slow muscle fibers with respect to intermediate and fast axial muscles in adult zebrafish in control conditions, and that only in fast muscles its level was increased upon exercise training and/or during nerve/muscle regeneration following injury [3].

### 1.1. Glutamate Transporters at the Neuromuscular Junction in Vertebrates

The precise localization of glutamate in motoneurons and specifically at presynaptic terminals of NMJs remains elusive, and its presence within synaptic vesicles has never been unambiguously demonstrated [2]. Data supporting the presence of vesicular glutamate transporters at the NMJ and cholinergic synapses are rather contradictory [1,4,5,6,7,8]. Finally, besides the vesicular glutamate transporters, other molecules involved in handling glutamate at central synapses, namely glial glutamate transporters, are present at the NMJ in rodents, supporting the notion that glutamate is released at the motor endplate.

### 1.2. Vesicular Glutamate Transporters

Three main types of vesicular glutamate transporters are expressed in the excitatory glutamatergic synapses in the central nervous system (CNS) of vertebrates (VGLUTs 1–3); these transporters fill synaptic vesicle with the neurotransmitter thanks to the proton gradient across the vesicle membrane. Evidences showing the presence of vesicular glutamate transporters at NMJs are rather scanty and somehow contradictory: Herzog and colleagues demonstrated that rat spinal motoneurons express the vesicular glutamate transporters 1 and 2 (VGLUT1 and VGLUT2) but these are confined at the central nerve terminals that contact Renshaw inhibitory interneurons and they are not found at neuromuscular synapses [5]. Indeed, at central synapses, mammalian motoneurons corelease ACh and glutamate [7].

Conversely, NMJs immunoreactive for VGLUT1 were found in mouse striated esophageal muscles [6,9], while NMJs of other somitic and branchiogenic muscles (i.e., soleus, tibilias anterior, masseter) were negative for all the three isoforms of the transporter [6]. Recently, VGlut1 (but not VGlut2) positive puncta, in close proximity to vesicular ACh transporter (VAChT) positive areas, were observed in adult zebrafish motoneurons innervating the lateral axial muscle [3].

On the other hand, it was shown that VGLUT3 was present in adult rat skeletal muscle homogenates, and VGLUT3 like-immunoreactivity was found at the NMJs of the soleus, close to both ACh vesicular transporter (VAChT) positive areas and the ACh receptor clusters [4]. Strikingly, the coexistence of VGLUT1 and 2 and of the VAChT (and of nucleotide transporters) was reported on a high proportion of synaptic vesicles from the torpedo electric organ, a structure that is considered a classical model of cholinergic neurotransmission [8].

All these evidences about central synapses and NMJs suggest that multiple neurotransmitters can be co-released by motoneurons and that glutamate can play a relevant role as a modulator of synaptic transmission and plasticity, as it does in the CNS (reviewed in [10]). However, the strongest evidences of glutamate corelease at the NMJ, to enhance/modulate the motor output, only derive from the recent studies on adult zebrafish NMJs [3].

While all the above-mentioned reports indicate or suggest that glutamate is metabolized within the presynaptic terminals of NMJs (as in central synapses), there is an alternative hypothesis about its origin at the motor endplate. Indeed, the presence of *N*-acetyl aspartyl glutamate (NAAG) at the vertebrate diaphragm NMJ has been documented since 1995 [11], and more recently the role of this peptide neurotransmitter at the NMJ has been clarified. It was shown that NAAG is present within the presynaptic terminals in the lizard ceratomandibularis NMJs and that it is released from the nerve endings upon depolarization. After its release into the synaptic cleft, NAAG directly binds and activates presynaptic group-III glutamate metabotropic receptors (mGluR3, see below) and/or it is hydrolyzed by specific glutamate peptidases (GPII) to *N*-acetyl aspartate (NAA) and glutamate; the latter is able to bind and activate other metabotropic (mGluR2) and ionotropic (NMDA) receptors expressed there [12]. It has to be noted, however, that the two possible sources of glutamate can easily coexist at the NMJ as they co-exist in many glutamatergic central synapses [12] (and references therein).

### 1.3. Excitatory Amino Acids Transporters

Excitatory amino-acids transporters (EAATs) are membrane proteins that, besides glutamate, bind and transport aspartate. The function of these molecules is to terminate the excitatory signal at synapses by rapidly removing glutamate from the synaptic cleft. The activity of these high-affinity transporters is dependent on the electrochemical gradient of Na^+^; in the CNS, they play a fundamental role in the regulation of the concentrations of glutamate in the synaptic cleft and in the extracellular space to keep glutamate concentration below excitotoxicity level. Five subtypes of glutamate transporters have been identified in rodents and humans and named EAAT1–5 (reviewed in [13]). Subtypes EAAT1–2 are found in membranes of glial cells (astrocytes, microglia, and oligodendrocytes, and named GLAST-1 and GLT-1, respectively, in rodents) and in endothelial cells, while EAAT3–5 are expressed in neurons. The glial transporters—in particular the various splice variants of GLT-1 (EAAT2)—have a predominant role in the re-uptake of glutamate from the extracellular space. While there are no data about the presence of the neuronal transporter subtypes at the NMJ, GLAST-1 and GLT-1 are found in rat skeletal muscle-nerve homogenates [14], and they are localized on the postsynaptic membrane of EDL and soleus NMJs [15] and in perisynaptic Schwann cells [16]. GLAST-1 is also present at the NMJ of the frog cutaneous pectoris muscle, where it co-localizes with markers of terminal Schwann cells [17].

### 1.4. Ionotropic and Metabotropic Glutamate Receptors

Ionotropic glutamate receptors are channel receptors that differ in their permeability to cations, selectivity, activation/inactivation kinetics, desensitization properties, and pharmacology. In vertebrates, ionotropic receptors have been classified into three main classes according to their selective agonist: (1) α-amino-3-idroxy-5-methyl-4-isoxasol-propionic acid (AMPA) receptors; (2) kainate receptors; (3) *N*-methyl-d-aspartate (NMDA) receptors—and a small group of orphan receptors δ1 and δ2. Most AMPA (those containing the GluA2 subunit) and kainate receptor-channels are permeable to monovalent ions (Na^+^ and K^+^), share several kinetic and pharmacological properties, and the former specifically mediates fast synaptic transmission. NMDA receptors, instead, are permeable to monovalent but also divalent cations (i.e., Ca^2+^) and are mostly involved in the modulation of synaptic responses and in plasticity.

To date, eight cDNAs coding for metabotropic glutamate receptor subunits have been identified in vertebrates (mGluR1 to mGluR8) and, based on their structure, activation of downstream signaling pathways, and pharmacology, these have been subdivided in three main classes: (1) Group I—mGluR1 and mGluR5 are coupled to Gq proteins that activate phospholipase C and the downstream signaling cascade that involves inositol trisphosphate and the intracellular Ca^2+^ mobilization from intracellular stores; (2) Group II (mGluR2 and mGluR3) and (3) Group III (mGluR4 and mGluR6–8) are coupled to Gi whose activation inhibits adenylate cyclase. Diverse types of mGluRs are differently distributed in cells and, in particular at the synapse, they can be localized either in the post- or in the pre-synaptic membranes, where they play a role in regulating the activity of ionotropic receptors but also in synaptic plasticity.

### 1.5. Ionotropic and Metabotropic Glutamate Receptors at the Neuromuscular Junction in Vertebrates

In vertebrates, the glutamate released at the NMJ modulates synaptic transmission through its interaction with presynaptic receptors, but also through the activation of both ionotropic and metabotropic postsynaptic receptors.

#### 1.5.1. Presynaptic Glutamate Receptors

Glutamate at the NMJ was shown to act on the modulation of cholinergic synaptic transmission through its interaction with presynaptic receptors. At the NMJ of tail muscles of *Xenopus laevis* embryos, glutamate activates presynaptic ionotropic receptors of the kainate/AMPA and NMDA type. Activation of these receptors induces an increase in the spontaneous release of ACh [18]. The effect of glutamate on the increased spontaneous release of ACh is mediated by the influx of Ca^2+^ that occurs through the NMDA receptors themselves and through the opening of the voltage-activated l-type Ca^2+^ channels. Strikingly, this effect also occurred in young tadpoles while it was absent in mature tadpoles and in adult frogs [19]. Similarly, in larval and adult zebrafish NMJs, the activation of the ionotropic presynaptic glutamate receptors resulted in a significant increase of the spontaneous ACh release [3,20]. The presence of ionotropic glutamate receptors at the NMJ of the zebrafish larvae was assessed by means of electrophysiological, biochemical, and immunofluorescence experiments, while their characterization was achieved through pharmacological inhibition. All these assays indicated the presence of the AMPA, but not the kainate type of receptor, and of the NMDA receptor containing the GluN2A subunit [20]. The same methodological approaches were used for the identification of the NMDA receptor containing the GluN2A and B subunits in motor axons and NMJ presynaptic terminals, respectively, in adult zebrafish [3].

In those experimental models, the activation of glutamate ionotropic receptors in the presynapses and the resulting Ca^2+^ influx played a pivotal role in the positive modulation of the activity of the developing NMJ and thus in the pre- and post-synaptic developmental changes that occur during embryonic and larval neuromuscular apparatus maturation.

It is worth mentioning that, with the exception of the zebrafish model [3], the potentiating effect of glutamate on ACh release at the vertebrate NMJ in embryos and larvae tend to be substituted with an inhibitory one later in life, when glutamate prevalent action is dampening of synaptic activity. This effect is most likely mediated by the activation of presynaptic metabotropic glutamate receptors (mGluRs). In fact, mGluRs are present in nerve endings of NMJ of the adult lizard and frog muscles [12,21]. The presence of mGluR3 and mGluR1a/5 was demonstrated by means of immunofluorescence experiments; in both experimental models, the activation mGluRs reduces the level of spontaneous [12,21] and evoked ACh release [21] (Table 1).

#### 1.5.2. Postsynaptic Glutamate Receptors

Glutamate released at the NMJ in vertebrates can exert its effects through the activation of postsynaptic NMDA receptors (containing the GluN1 subunit) whose presence in the postsynaptic membranes of skeletal muscle was first reported in the rat diaphragm in 1995 [11]. Few years later, this observation was confirmed and it was demonstrated that, in rodent NMJs from type II (fast twitch) muscles, NMDAR-1 colocalized with nitric oxide synthase I [22]. More recent analyses have shown that GluN1 containing NMDA receptors are present on the postsynaptic membrane of NMJs from slow (soleus), fast (EDL), and mixed (diaphragm) muscles and that they are localized within the depth of the secondary folds [23]. Recently, GluN1 containing NMDA receptors on the postsynaptic membrane of adult frog cutaneous pectoris NMJ were shown [21]. Postsynaptic glutamate receptors are activated by endogenous glutamate at the NMJ upon moderate/high frequency stimulation [24,25] (and references therein).

Nitric oxide (NO) acts as a rapid diffusible retrograde messenger from muscle to nerve. In many vertebrates, the NO synthetic enzyme, nitric oxide synthase, is associated with the NMJs and with the postsynaptic membranes of skeletal muscle fibers [26] (and references therein). Glutamate may modulate synaptic efficacy at the NMJ via its effect on the synthesis of NO; in fact, glutamate binding to NMDA receptors at the postsynaptic membrane activates cation-selective ion channels and generates a Ca^2+^ influx that, in turn, activates nitric oxide synthase and the NO cascade. Several experimental lines of evidence in vitro and in vivo indicate that, in the presynaptic terminals of active NMJs, NO negatively modulates neurotransmitter release [27] (and references therein) and also inhibits the non-quantal release of acetylcholine from nerve endings in the rat diaphragm [28,29,30,31]. The negative modulation of neurotransmitter release by NO (through the activation of guanylate-cyclase and increased levels of cyclic GMP (cGMP)) is linked, in particular, to inactivating phosphorylation(s) of voltage-dependent Ca^2+^ channels at the presynaptic active zone. The Ca^2+^ influx through these channels is necessary to trigger the evoked quantal release and, thus, the generation of evoked end-plate potential [32]. Pre-synaptic cGMP can also upregulate miR-124 and miR-142 expression leading to the downregulation of Rab3a mRNA and protein level and consequent reduction of ACh quantal release at the hindlimb NMJ of adult mice [33].

It was also shown that, in the same experimental model, 24-hydroxycholesterol (24S-HC) administration counteracts the negative effects of NMDA-receptor activation on neurotransmitter release. Indeed, during evoked release, 24S-HC downregulated post-synaptic eNOS activity, leading to the suppression of NO/cGMP signaling pathway and to the increase of synaptic vesicle exocytosis and recycling [24]. Strikingly, 24S-HC seemed to play a different role in amyotrophic lateral sclerosis (ALS) pathogenesis. In SOD1^G93A^ mice, the 24S-HC mediated NO downregulation led to a decreased vesicle exocytosis, an opposite effect compared to healthy mice. Indeed, mutant mice were characterized by an upregulation of activity-induced NO synthesis and this was probably due to altered lipids rafts dynamics [34]. These observations suggest that membrane microdomains and lipid rafts dynamics might play an important role in neuronal signaling (reviewed in [35,36]) also at the NMJ.

Neuronal excitability can be modulated by NO in a cGMP-independent manner by means of S-nitrosylation of target substrates like ion channels and ligand gated receptors (reviewed in [37]). Even though this effect has never been investigated in detail in vertebrate NMJs, recent evidence from the *Drosophila* NMJ suggests that NO can directly influence nitrosylation of the presynaptic protein complexin (cpx), thus reducing its farnesylation. Complexin is a fusion-clamp protein needed to modulate synaptic vesicle release. Farnesylated complexin is targeted to endomembranes while the non-farnesylated protein remains in the cytosol where it competes with synaptotagmin for the binding to the soluble NSF attachment protein receptor (SNARE) complex, resulting in a reduction of neurotransmitter release [38]. Given that at mouse EDL NMJs, Complexin-1 is necessary to positively modulate and to synchronize vesicle exocytosis, possibly by favoring the proximity of Ca^2+^ channels with the exocytic machinery [39], it is then possible to hypothesize that NO-induced S-nitrosylation could affect Cpx localization and/or function, altering synaptic vesicle exocytosis also at the vertebrate NMJ.

A further action of NO at the NMJ is the activation of a guanylate-cyclase and the increase of intracellular levels of cGMP within muscle fibers. Increased cGMP activates protein kinases that phosphorylate membrane proteins (channels and/or transporters), and contributes to the maintenance of the resting membrane potential of rat diaphragm muscle fibers [40]. Among the targets of these kinases, there is the furosemide-sensitive Cl^−^ transporter, whose phosphorylation is important to maintain the transporter in an inactive state. When glutamate signaling from the nerve ending is missing (alongside the non-quantal release of ACh), like in denervated fibers, the signaling cascade is interrupted, the dephosphorylated transporter is active, and Cl^−^ anions enter the fiber that undergoes depolarization with a reduction of its resting membrane potential [31,40,41].

It has been recently demonstrated that postsynaptic NMDA receptor activation at mouse hindlimb NMJ plays a fundamental role in contributing to the elimination of excess synapse input during early postnatal life by stabilizing stronger synapses and destabilizing the weakest. This effect is once again associated with the NMDA-dependent increase of Ca^2+^ within the muscle fiber [42] and suggests that, also in this peripheral synapse, NMDA receptors may function as coincidence detector. Coincidence detection through NMDA activation is needed for synaptic plasticity in the CNS [43] (and references therein), and thus, possibly for neuromuscular innervation refinement during early postnatal life in rodents. Whereas the signaling pathways triggered by the NMDA-dependent Ca^2+^ influx into central postsynaptic neurons leading to plasticity are mostly known, the mechanisms driving input selection at the developing neuromuscular apparatus await elucidation.

Along with the presence of the GluN1 and GluN2A subunits of the NMDA glutamate receptor, the presence of the subunits GluA1 and GluA2/3 of the AMPA receptor subtype have been documented in the postsynaptic membrane of NMJs in quadriceps muscle of young mice; the precise function of these AMPA receptor is, to date, unknown [44].

In the frog NMJ, endogenous glutamate reduces—in a frequency dependent manner—the probability of acetylcholine release without interfering with the extracellular Ca^2+^ entry into presynaptic terminals. This effect is mediated by the activation of mGluRs that are localized at the endplate, clustered in the postsynaptic membranes and interspersed among the clusters of acetylcholine receptor [17]. Interestingly, the chemical mediator that is responsible for mGluR-dependent synaptic transmission depression upon stimulation with glutamate, is once again NO. As the activation of NO synthase is dependent on increased level of intracellular Ca^2+^, mGluRs activation must be linked to and mobilize this cation from intracellular Ca^2+^ stores (ryanodine-sensitive or IP_3_-sensitive Ca^2+^ stores) in muscle fibers, either through a direct interaction with scaffold proteins or through the production of IP_3_ that will then mobilize Ca^2+^ from IP_3_-sensitive stores [45] (and references therein). The presence of group II mGluRs (mGluR2) at the postsynaptic membrane was also shown at the lizard NMJ [12].

**Figure 1:**
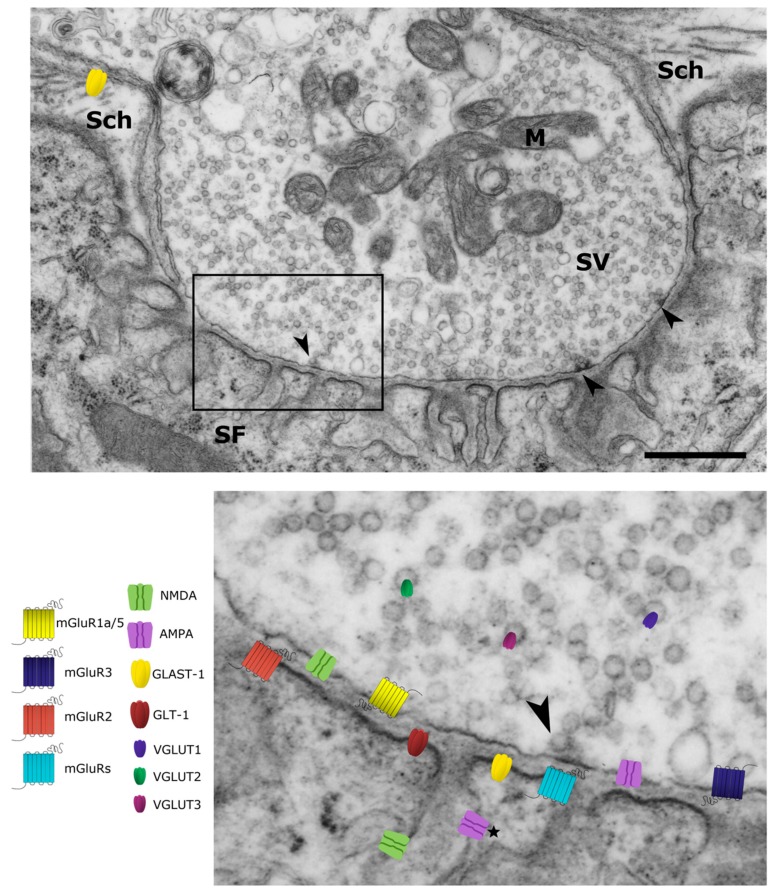
Glutamate signaling machinery at the vertebrate neuromuscular junction. Low magnification image of a thin section of a synaptic bouton from mouse diaphragm, the presynaptic terminal is filled with synaptic vesicles (SV) and mitochondria (M); active zones are indicated (Arrowheads). The presynaptic terminal lies in a shallow primary fold that is lined with secondary folds (SF). The whole structure is covered by Schwann cell processes (Sch) (Scale bar 1 μm). In the inset, the localization of glutamate receptors and transporters is summarized. This simplified scheme recapitulates the observations derived from neuromuscular junctions (NMJs) of different vertebrate species at different ages. The position of receptor subtypes and transporters on the image reflects their subcellular localization as defined by the experimental data reported in the cited papers (see text and Table 1). Noteworthy, whereas the presence of *N*-methyl-d-aspartate (NMDA) receptors within secondary folds has been demonstrated at the ultrastructural level [23], the presence of α-amino-3-idroxy-5-methyl-4-isoxasol-propionic acid (AMPA) receptors in the same compartment is only putative (*).

The occurrence of all these molecules involved in glutamate handling at the NMJ of vertebrates (summarized in Table 1 and Figure 1) is suggestive of the presence of a functional glutamatergic system whose role might be broader than that of mere modulation of the ACh neurotransmission. Given that motoneurons synthesize and release glutamate as neurotransmitter at their central synapses [5,7], it is possible to hypothesize that glutamate could play that role also at the NMJs, under those circumstances requiring plastic remodeling and synaptic refinement (i.e., during neuromuscular innervation development and maturation, and during increased locomotor activity or following traumatic injury).

## 2. Do Glutamatergic Neuromuscular Junctions Exist in Vertebrates?

Although it has long been assumed that neurotransmission at the NMJ in vertebrates is always and only cholinergic, recent evidences have challenged this notion and suggested that other neurotransmitters might play a role here broader than expected.

### 2.1. Glutamate as a Neurotransmitter at the Neuromuscular Junction during Embryonic Development of Vertebrates

During a critical developmental window in the embryonic neuronal development, the neurotransmitter phenotype selection in vertebrate motoneurons depends on specific oscillations of calcium spikes. Any perturbation, induced with genetic or pharmacological tools, of these endogenous calcium oscillations results in a shift from a cholinergic phenotype to the generation of synapses that contain other neurotransmitters; in particular enhancement of calcium spikes in *Xenopus* spinal neurons induce a shift toward the expression of inhibitory neurotransmitters (i.e., glycine and γ-aminobutyric acid, GABA) while downregulation of activity results in an increased production of excitatory neurotransmitters (i.e., ACh and glutamate). Interestingly, in this experimental paradigm, each individual neuron can synthesize a single neurotransmitter (ACh or glutamate), while others can synthesize and corelease several mediators [46,47].

During early embryonic development, the muscle itself expresses mRNA and receptor proteins for several neurotransmitters including ACh, glutamate, GABA, and glycine receptors; all but ACh receptor mRNAs and proteins are subsequently eliminated to achieve the cholinergic phenotype of the mature NMJ. In vitro experiments have shown that by modifying the neurotransmitter of motoneurons, as indicated above, it is possible to alter the receptor expression profiles on the muscle fibers in order to ensure the correct transmitter–receptor matching at the NMJ [48]. This activity-dependent transmitter–receptor matching at synapses has obvious relevant implications in the study of the development of the central and peripheral nervous system in physiological conditions but also has functional implications for neuronal circuitry repair in the mature nervous system [49,50] (and references therein).

### 2.2. Glutamate Is the Neurotransmitter Operating at the Neuromuscular Junction in an Experimental Model of Spinal Cord Injury

In an experimental model of spinal cord injury in rats, connecting the distal stump of the transected nerve of abdominal muscles with the lateral bundle of rat spinal cord, by a peripheral nerve graft, produces functional muscle reinnervation. Reinnervation occurs by axonal elongation of glutamatergic supraspinal neurons originating from rubrospinal, reticulospinal, and vestibulospinal tracts [51]. Reinnervated muscle becomes insensitive to classical nicotinic receptor antagonists but sensitive to selective blockers of glutamate AMPA receptor [51,52]. In this experimental model of spinal cord injury, where a switch from a cholinergic to a glutamatergic input occurs, the postsynaptic cholinergic apparatus does not disappear nor change its architecture, but AMPA-type ionotropic glutamate receptors are synthesized, or more likely are upregulated and inserted into the postsynaptic plasma membrane where they colocalize with the ACh receptor clusters. Indeed, not only the clusters of ACh and AMPA receptors colocalize by immunofluorescence, but also their scaffolding proteins (rapsyn and stargazin, respectively) coimmunoprecipitate [14].

Grafted nerve expresses high levels of the vesicular glutamate transporters VGLUT1 and VGLUT2 [51] and about 30% of the presynaptic terminals of the NMJs in these reinnervated muscles are strongly VGLUT2 immunoreactive. Immunogold experiments at the ultrastructural level corroborated the evidence that in the presynaptic terminals of reinnervated NMJs, the glutamate content is significantly higher than in contralateral cholinergic endplates; moreover, stereological analysis demonstrated that glutamate immunoreactivity is associated with the synaptic vesicle pool. The electron microscopy immunolocalization of glutamate, together with the presence of the vesicular transporter VGLUT2 in presynaptic terminals, suggest that in NMJs reinnervated by axons of excitatory glutamatergic central neurons, glutamate is contained in synaptic vesicles and might be released upon stimulation in a quantal manner [14]. These studies also demonstrated that adult rat motor endplates, already committed to receive a cholinergic input and expressing high levels of clustered acetylcholine receptors, nonetheless retain the ability to shift from a cholinergic to a glutamatergic phenotype in response to presynaptic glutamatergic input in order to guarantee the appropriate transmitter–receptor matching.

## 3. Conclusions

Across phyla, glutamate and ACh are by far the most common neurotransmitters used at central excitatory synapses and at NMJs. Glutamate is the main neurotransmitter at the neuromuscular junction of insects and crustaceans, but its role in neuromuscular transmission has also been demonstrated in organisms belonging to different phyla. Interestingly, while in mollusks the great majority of motoneurons are cholinergic, in the snail *Aplysia*, a subset of buccal neuromuscular synapses use glutamate for fast neurotransmission [53]. The existence of these glutamatergic endplates in organisms whose neuromuscular transmission is mostly cholinergic is very striking and these evidences seem to indicate that, at a certain point during invertebrate evolution, the selection of the prevalent chemical mediator used at the NMJ was an open option.

During embryonic and early postnatal development in fish, amphibians, and mammals, glutamate plays an important role as a positive modulator of cholinergic neurotransmission and synaptic plasticity as, at least in mammals, it represents a key molecule involved in the regulation of excess synapse elimination and stabilization of the stronger synaptic inputs on muscle fibers by means of the activation of a plasticity related receptor like the NMDA receptor on the postsynaptic membrane. These observations suggest that glutamate can be release together with ACh and they also suggest that corelease of multiple neurotransmitters at this peripheral synapse can be a widespread feature, at least during development. Strikingly, it has been demonstrated that, during embryonic development, it is possible to experimentally modify the chemical nature of the neurotransmitters released at the motor endplate and that, when this shift occurs, the muscle fibers react to this respecification by synthesizing the matching receptors that are then inserted and clustered in the postsynaptic membrane.

The recent discovery of glutamate corelease at adult zebrafish NMJs and its specific upregulation in fast muscles neurotransmission upon exercise training and during regeneration following injury, are suggestive of a role of glutamate as a mediator of synaptic plasticity and indicate that some sort of dynamic and reversible neurotransmitter respecification to enhance motor function can occur also in adult organisms.

In vertebrates, motoneurons corelease acetylcholine and glutamate at their central synapses and the notion that glutamate is also released at the NMJ is further supported by the observation that all the molecules involved in the handling of glutamate at central synapses (transporters and receptors) are present at the motor endplate and that many of the molecules that are involved in the downstream effects of glutamate signaling at central synapses are also present at the NMJ. Despite the diversity of ionotropic and metabotropic glutamate receptors found in central synapses, however, it is worth to note that, to date, only a subset of receptor subtypes was identified at the NMJ and that their role and the signal transduction cascade involved in glutamate signaling here deserve further investigation.

In adult rodents, axons from glutamatergic supraspinal neurons can grow in a peripheral nerve graft and make functional synapses with denervated skeletal muscle fibers. These reinnervated NMJs are glutamatergic and the reinnervated muscle fibers express higher levels of ionotropic glutamate AMPA receptors that are inserted in the postsynaptic plasma membrane where they co-cluster with the ACh receptors.

Taken together, these studies demonstrate that, in some circumstances, such as when plastic remodeling of the neuronal input is needed or when motor functions have to be restored or enhanced, it is possible to have glutamate neurotransmission at NMJs in vertebrates and that the nervous input can also affect the type of neurotransmitter receptors in the postsynaptic membrane in adult organisms.

## Figures and Tables

**Table 1:** Glutamate signaling machinery at the vertebrate neuromuscular junction.

Model NMJ	Target Molecule	Localization	Refs.
Adult rat	Glutamate	Presynaptic	[1,2]
Frog embryo and tadpole	Ionotropic glutamate receptor—kainate/AMPA and NMDA	Presynaptic	[19]
Zebrafish embryo and adult	Ionotropic glutamate receptor—AMPA and GluN2A and B containing NMDA	Presynaptic	[9,20]
Adult lizard and frog	Metabotropic glutamate receptor—mGluR3 and mGluR1a/5	Presynaptic	[12,21]
Adult frog, mouse and rat	Ionotropic glutamate receptor—GluN1 and/or GluN2 containing NMDA	Postsynaptic	[11,21,22,23,44]
Adult mouse	Ionotropic glutamate receptor—GluA1 and GluA2/3 containing AMPA	Postsynaptic	[44]
Adult lizard and frog	Metabotropic glutamate receptors (unspecified) and mGluR2	Postsynaptic	[12,17]
Adult mouseesophageal muscle	Vesicular glutamate transporter—VGLUT1	Presynaptic	[5,8]
Adult zebrafish	Vesicular glutamate transporter—VGlut1	Presynaptic	[9]
Adult rat	Vesicular glutamate transporter—VGLUT3	Presynaptic	[3]
Torpedo electric organ	Vesicular glutamate transporter—VGlut1 and VGlut2	Presynaptic	[7]
Adult rodent	High affinity transporter for excitatory amino acids, GLAST-1 and GLT-1	Postsynaptic and glial cell processes	[14,15,16]
Adult frog	High affinity transporter for excitatory amino acids, GLAST-1	Terminal Schwann cells	[17]

## References

[1] Meister B., Arvidsson U., Zhang X., Jacobsson G., Villar M.J., Hökfelt T. (1993). Glutamate transporter mRNA and glutamate-like immunoreactivity in spinal motoneurones. Neuroreport.

[2] Waerhaug O., Ottersen O.P. (1993). Demonstration of glutamate-like immunoreactivity at rat neuromuscular junctions by quantitative electron microscopic immunocytochemistry. Anat. Embryol. (Berl.).

[3] Bertuzzi M., Chang W., Ampatzis K. (2018). Adult spinal motoneurons change their neurotransmitter phenotype to control locomotion. Proc. Natl. Acad. Sci. USA.

[4] Boulland J.L., Qureshi T., Seal R.P., Rafiki A., Gundersen V., Bergersen L.H., Fremeau R.T., Edwards R.H., Storm-Mathisen J., Chaudhry F.A. (2004). Expression of the vesicular glutamate transporters during development indicates the widespread corelease of multiple neurotransmitters. J. Comp. Neurol..

[5] Herzog E., Landry M., Buhler E., Bouali-Benazzouz R., Legay C., Henderson C.E., Nagy F., Dreyfus P., Giros B., El Mestikawy S. (2004). Expression of vesicular glutamate transporters, VGLUT1 and VGLUT2, in cholinergic spinal motoneurons. Eur. J. Neurosci..

[6] Kraus T., Neuhuber W.L., Raab M. (2004). Vesicular glutamate transporter 1 immunoreactivity in motor endplates of striated esophageal but not skeletal muscles in the mouse. Neurosci. Lett..

[7] Nishimaru H., Restrepo C.E., Ryge J., Yanagawa Y., Kiehn O. (2005). Mammalian motor neurons corelease glutamate and acetylcholine at central synapses. Proc. Natl. Acad. Sci. USA.

[8] Li H., Harlow M.L. (2014). Individual synaptic vesicles from the electroplaque of Torpedo californica, a classic cholinergic synapse, also contain transporters for glutamate and ATP. Physiol. Rep..

[9] Ewald P., Neuhuber W.L., Raab M. (2006). Vesicular glutamate transporter 1 immunoreactivity in extrinsic and intrinsic innervation of the rat esophagus. Histochem. Cell Biol..

[10] Trudeau L.E., El Mestikawy S. (2018). Glutamate Cotransmission in Cholinergic, GABAergic and Monoamine Systems: Contrasts and Commonalities. Front. Neural Circuits.

[11] Berger U.V., Carter R.E., Coyle J.T. (1995). The immunocytochemical localization of *N*-acetylaspartyl glutamate, its hydrolysing enzyme NAALADase, and the NMDAR-1 receptor at a vertebrate neuromuscular junction. Neuroscience.

[12] Walder K.K., Ryan S.B., Bzdega T., Olszewski R.T., Neale J.H., Lindgren C.A. (2013). Immunohistological and electrophysiological evidence that *N*-acetylaspartylglutamate is a co-transmitter at the vertebrate neuromuscular junction. Eur. J. Neurosci..

[13] Benarroch E.E. (2010). Glutamate transporters: Diversity, function, and involvement in neurologic disease. Neurology.

[14] Francolini M., Brunelli G., Cambianica I., Barlati S., Barbon A., La Via L., Guarneri B., Boroni F., Lanzillotta A., Baiguera C. (2009). Glutamatergic reinnervation and assembly of glutamatergic synapses in adult rat skeletal muscle occurs at cholinergic endplates. J. Neuropathol. Exp. Neurol..

[15] Rinholm J.E., Slettaløkken G., Marcaggi P., Skare Ø., Storm-Mathisen J., Bergersen L.H. (2007). Subcellular localization of the glutamate transporters GLAST and GLT at the neuromuscular junction in rodents. Neuroscience.

[16] Carozzi V.A., Canta A., Oggioni N., Ceresa C., Marmiroli P., Konvalinka J., Zoia C., Bossi M., Ferrarese C., Tredici G. (2008). Expression and distribution of ‘high affinity’ glutamate transporters GLT1, GLAST, EAAC1 and of GCPII in the rat peripheral nervous system. J. Anat..

[17] Pinard A., Lévesque S., Vallée J., Robitaille R. (2003). Glutamatergic modulation of synaptic plasticity at a PNS vertebrate cholinergic synapse. Eur. J. Neurosci..

[18] Fu W.M., Liou J.C., Lee Y.H., Liou H.C. (1995). Potentiation of neurotransmitter release by activation of presynaptic glutamate receptors at developing neuromuscular synapses of Xenopus. J. Physiol..

[19] Liou H.C., Yang R.S., Fu W.M. (1996). Potentiation of spontaneous acetylcholine release from motor nerve terminals by glutamate in Xenopus tadpoles. Neuroscience.

[20] Todd K.J., Slatter C.A., Ali D.W. (2004). Activation of ionotropic glutamate receptors on peripheral axons of primary motoneurons mediates transmitter release at the zebrafish NMJ. J. Neurophysiol..

[21] Tsentsevitsky A., Nurullin L., Nikolsky E., Malomouzh A. (2017). Metabotropic and ionotropic glutamate receptors mediate the modulation of acetylcholine release at the frog neuromuscular junction. J. Neurosci. Res..

[22] Grozdanovic Z., Gossrau R. (1998). Co-localization of nitric oxide synthase I (NOS I) and NMDA receptor subunit 1 (NMDAR-1) at the neuromuscular junction in rat and mouse skeletal muscle. Cell Tissue Res..

[23] Malomouzh A.I., Nurullin L.F., Arkhipova S.S., Nikolsky E.E. (2011). NMDA receptors at the endplate of rat skeletal muscles: Precise postsynaptic localization. Muscle Nerve.

[24] Kasimov M.R., Fatkhrakhmanova M.R., Mukhutdinova K.A., Petrov A.M. (2017). 24S-Hydroxycholesterol enhances synaptic vesicle cycling in the mouse neuromuscular junction: Implication of glutamate NMDA receptors and nitric oxide. Neuropharmacology.

[25] Slater C.R. (2015). The functional organization of motor nerve terminals. Prog. Neurobiol..

[26] Ribera J., Marsal J., Casanovas A., Hukkanen M., Tarabal O., Esquerda J.E. (1998). Nitric oxide synthase in rat neuromuscular junctions and in nerve terminals of Torpedo electric organ: Its role as regulator of acetylcholine release. J. Neurosci. Res..

[27] Worden M.K. (1998). Modulation of vertebrate and invertebrate neuromuscular junctions. Curr. Opin. Neurobiol..

[28] Mukhtarov M.R., Vyskocil F., Urazaev A.K., Nikolsky E.E. (1999). Non-quantal acetylcholine release is increased after nitric oxide synthase inhibition. Physiol. Res..

[29] Mukhtarov M.R., Urazaev A.K., Nikolsky E.E., Vyskocil F. (2000). Effect of nitric oxide and NO synthase inhibition on nonquantal acetylcholine release in the rat diaphragm. Eur. J. Neurosci..

[30] Malomouzh A.I., Mukhtarov M.R., Nikolsky E.E., Vyskocil F., Lieberman E.M., Urazaev A.K. (2003). Glutamate regulation of non-quantal release of acetylcholine in the rat neuromuscular junction. J. Neurochem..

[31] Vyskocil F., Malomouzh A.I., Nikolsky E.E. (2009). Non-quantal acetylcholine release at the neuromuscular junction. Physiol. Res..

[32] Adámek S., Shakirzyanova A.V., Malomouzh A.I., Naumenko N.V., Vyskočil F. (2010). Interaction of glutamate- and adenosine-induced decrease of acetylcholine quantal release at frog neuromuscular junction. Physiol. Res..

[33] Zhu H., Bhattacharyya B., Lin H., Gomez C.M. (2013). Skeletal muscle calpain acts through nitric oxide and neural miRNAs to regulate acetylcholine release in motor nerve terminals. J. Neurosci..

[34] Mukhutdinova K.A., Kasimov M.R., Giniatullin A.R., Zakyrjanova G.F., Petrov A.M. (2018). 24S-hydroxycholesterol suppresses neuromuscular transmission in SOD1(G93A) mice: A possible role of NO and lipid rafts. Mol. Cell Neurosci..

[35] Krivoi I.I., Petrov A.M. (2019). Cholesterol and the Safety Factor for Neuromuscular Transmission. Int. J. Mol. Sci..

[36] Egawa J., Pearn M.L., Lemkuil B.P., Patel P.M., Head B.P. (2016). Membrane lipid rafts and neurobiology: Age-related changes in membrane lipids and loss of neuronal function. J. Physiol..

[37] Ahern G.P., Klyachko V.A., Jackson M.B. (2002). cGMP and S-nitrosylation: Two routes for modulation of neuronal excitability by NO. Trends Neurosci..

[38] Robinson S.W., Bourgognon J.M., Spiers J.G., Breda C., Campesan S., Butcher A., Mallucci G.R., Dinsdale D., Morone N., Mistry R. (2018). Nitric oxide-mediated posttranslational modifications control neurotransmitter release by modulating complexin farnesylation and enhancing its clamping ability. PLoS Biol..

[39] Lin M.Y., Rohan J.G., Cai H., Reim K., Ko C.P., Chow R.H. (2013). Complexin facilitates exocytosis and synchronizes vesicle release in two secretory model systems. J. Physiol..

[40] Urazaev A.K., Naumenko N.V., Nikolsky E.E., Vyskocil F. (1999). The glutamate and carbachol effects on the early post-denervation depolarization in rat diaphragm are directed towards furosemide-sensitive chloride transport. Neurosci. Res..

[41] Vyskocil F. (2003). Early postdenervation depolarization is controlled by acetylcholine and glutamate via nitric oxide regulation of the chloride transporter. Neurochem. Res..

[42] Personius K.E., Slusher B.S., Udin S.B. (2016). Neuromuscular NMDA Receptors Modulate Developmental Synapse Elimination. J. Neurosci..

[43] Tabone C.J., Ramaswami M. (2012). Is NMDA receptor-coincidence detection required for learning and memory?. Neuron.

[44] Mays T.A., Sanford J.L., Hanada T., Chishti A.H., Rafael-Fortney J.A. (2009). Glutamate receptors localize postsynaptically at neuromuscular junctions in mice. Muscle Nerve.

[45] Pinard A., Robitaille R. (2008). Nitric oxide dependence of glutamate-mediated modulation at a vertebrate neuromuscular junction. Eur. J. Neurosci..

[46] Borodinsky L.N., Root C.M., Cronin J.A., Sann S.B., Gu X., Spitzer N.C. (2004). Activity-dependent homeostatic specification of transmitter expression in embryonic neurons. Nature.

[47] Spitzer N.C., Root C.M., Borodinsky L.N. (2004). Orchestrating neuronal differentiation: Patterns of Ca^2+^ spikes specify transmitter choice. Trends Neurosci..

[48] Borodinsky L.N., Spitzer N.C. (2007). Activity-dependent neurotransmitter-receptor matching at the neuromuscular junction. Proc. Natl. Acad. Sci. USA.

[49] Spitzer N.C., Borodinsky L.N. (2008). Implications of activity-dependent neurotransmitter-receptor matching. Philos. Trans. R. Soc. Lond. B Biol. Sci..

[50] Spitzer N.C. (2015). Neurotransmitter Switching? No Surprise. Neuron.

[51] Brunelli G., Spano P., Barlati S., Guarneri B., Barbon A., Bresciani R., Pizzi M. (2005). Glutamatergic reinnervation through peripheral nerve graft dictates assembly of glutamatergic synapses at rat skeletal muscle. Proc. Natl. Acad. Sci. USA.

[52] Pizzi M., Brunelli G., Barlati S., Spano P. (2006). Glutamatergic innervation of rat skeletal muscle by supraspinal neurons: A new paradigm in spinal cord injury repair. Curr. Opin. Neurobiol..

[53] Fox L.E., Lloyd P.E. (1999). Glutamate is a fast excitatory transmitter at some buccal neuromuscular synapses in Aplysia. J. Neurophysiol..

